# Acute Pancreatitis Induced by Diabetic Ketoacidosis with Major Hypertriglyceridemia: Report of Four Cases

**DOI:** 10.1155/2020/7653730

**Published:** 2020-04-02

**Authors:** Karama Bouchaala, Mabrouk Bahloul, Sabrine Bradii, Hela Kallel, Kamilia Chtara, Mounir Bouaziz

**Affiliations:** Department of Intensive Care, HabibBourguiba University Hospital, Sfax, Tunisia

## Abstract

Acute pancreatitis (AP) is a real clinical challenge. Acute pancreatitis remains a common cause of emergency department consultations and a major cause for hospitalization. Gallstones and drinking a lot of alcohol are the most frequent causes of AP. Moreover, AP can be induced by diabetic ketoacidosis (DKA) complicated by hypertriglyceridemia. We report 4 cases of DKA with hypertriglyceridemia complicated by AP in previously undiagnosed diabetes patients. All of our patients presented to the emergency ward with abdominal pain. Their physical exam showed epigastric tenderness. An abdominal CT scan was performed for each patient, showing an AP grade E. Laboratory samples showed high serum glucose levels. They had metabolic acidosis with elevated anion gap. They had high lipasemia and amylasemia. Their lipid panel was disturbed with a high level of cholesterol (from 12.8 mmol/l to 33 mmol/l) and triglyceridemia (from 53 to 133 mmol/l). Our patients were admitted into our ICU where they received fluid resuscitation and intravenous insulin, and their triglycerides rates decreased gradually. Two patients recovered to a good health state, and the two others developed septic shock, requiring the use of large-spectrum antibiotics, and acute kidney injury (AKI) with refractory metabolic acidosis, requiring hemodialysis. Despite the intensive treatment, they developed an unrecoverable multiorgan failure. Through our case series, we aim to highlight the importance of making an early diagnosis, which can be difficult in some situations due to overlapping signs; however, it is crucial for a good recovery. A good understanding of the pathway of hypoinsulinemic states causing hypertriglyceridemia then AP is important because it is the key to best management.

## 1. Introduction

Acute pancreatitis (AP) is a real clinical challenge. Nowadays, acute pancreatitis represents a common cause of emergency department consultations and a major cause for hospitalization. Gallstones and drinking a lot of alcohol are the most frequent causes of AP [1]. Moreover, AP can be induced by diabetic ketoacidosis (DKA), complicated by hypertriglyceridemia [2]. However, and to the best of our knowledge, the association of this triad of pancreatitis, hypertriglyceridemia, and diabetic ketoacidosis and its treatment has not been widely discussed in the literature in both adults and children [3]. Rapid diagnosis is difficult due to overlapping signs, but it is crucial for adequate management. We report 4 cases of DKA with hypertriglyceridemia complicated by AP in previously undiagnosed diabetes patients.

## 2. Case Reports

### 2.1. Case 1

A previously healthy 12-year-old girl, with a 10-day history of nausea, polyuria, and polydipsia, was brought to the emergency department for abdominal pain and vomiting. The patient did not have any history of alcohol consumption or medical health problems besides this presenting complaint. She was admitted in the pediatric unit. On admission to the ICU, the physical exam showed diffuse abdominal pain. She had sunken eyes with a clear acetone-smelling breath.

Her blood pressure was at 80/40 mmHg, pulse at 140 beats/min, respiration rate at 35/min, pulse oxygen saturation (SpO_2_) under air room at 96%, and body temperature at 38°C. Laboratory samples showed serum glucose level at 24 mmol/l, pH at 7.19, bicarbonate at 10 mmol/l, anion gap at 26, lipase at 260 U/l, amylase at 244 U/l, cholesterol at 33 mmol/l, and serum triglyceride = 133 mmol/l. WBC count was at 14650/mm^3^ and glycated hemoglobin was at 11.0%. Aspartate aminotransferase (AST), alanine aminotransferase (ALT), alkaline phosphatase, and bilirubin values were normal.

**Figure 1:**
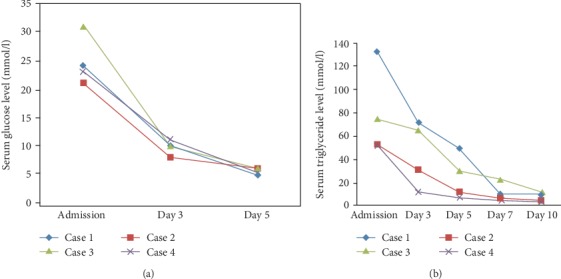
Evolution of serum glucose (a) and serum triglyceride (b) and level under treatment in all patients.

The abdominal ultrasound exam was normal. An abdominal CT scan was performed showing an AP grade E with three fluid collections and the presence of retroperitoneal air. The diagnosis of AP induced by DKA with concurrent hypertriglyceridemia was obtained, and the patient was transferred to our ICU for an adequate management. Continuous insulin infusion and fluid administration were initiated. The patient's health state improved. Her triglyceride rate decreased gradually (Figure 1). Continuous insulin was switched to subcutaneous insulin, and the patient was discharged after five days.

### 2.2. Case 2

A 12-year-old female child with no medical history and no history of prior biliary colic or alcohol consumption was admitted in the pediatric unit for abdominal pain and fever. Questioning the parents' revealed that, for 4 weeks, she had been suffering from polyuria and polydipsia associated with a significant loss of weight. The patient's health status deteriorated gradually, with abdominal pain and fever. The physical exam showed diffuse abdominal tenderness with an important decrease of bowel sounds. Her blood pressure was at 60/30 mmHg, pulse at 160 beats/min, temperature at 39°C, and respiratory rate at 30/min. The laboratory exams showed serum glucose level at 21 mmol/l, hemoglobin A1c at 16.5%, pH = 7.24, bicarbonate at 5 mmol/l, anion gap at 36, serum sodium = 142 mmol/l, serum potassium = 2.7 mmol/l, lipase = 411 U/l, amylase = 361 U/l, cholesterol = 12.8 mmol/l, and serum triglyceride = 53 mmol/l with apparent lipema in the lab test tubes. The other biochemical values were at the normal range, particularly the aspartate aminotransferase (AST), alanine aminotransferase (ALT), alkaline phosphatase, and bilirubin values.

Computed tomography (CT) scan revealed gastric distension, changes in the interstitium of the pancreas, and intrahepatic bile duct dilatation. The imaging did not show any other anomaly causing acute pancreatitis, in particular, a gallstone disease or extrahepatic duct dilatation. The patient developed respiratory distress needing the use of mechanical ventilation, then she was transferred to the intensive care unit. In ICU, she underwent fluid infusion, catecholamine prescription, and insulin infusion. During her ICU stay, she developed septic shock, requiring large-spectrum antibiotics, and acute kidney injury (AKI) with refractory metabolic acidosis, requiring hemodialysis. Despite the intensive treatment, the patient developed a multiorgan failure. She died 12 days after ICU admission.

### 2.3. Case 3

A 37-year-old male presenting with 2 weeks of polyuria, polydipsia, and epigastric pain and vomiting was admitted in an intensive care unit for consciousness alteration. Taking his medical history by interrogating his parents did not reveal any alcohol abuse or a prior history of biliary colic.

On admission, the initial assessment showed a Glasgow Coma Scale Score at 8/15, body temperature at 38.3°C, heart rate at 120 beats/min, blood pressure at 80/45 mmHg, respiratory rate at 30 breaths/min, and an oxygen saturation of 93% under room air. He needed intubation and mechanical ventilation.

The laboratory tests showed serum glucose at 32 mmol/l, pH = 7.31, bicarbonate = 10.7 mmol/l, serum sodium = 132 mmol/l, serum potassium = 3.9 mmol/l, lipase = 511 U/l, cholesterol = 12.8 mmol/l, serum triglyceride = 75 mmol/l, WBC count = 3900/mm^3^, platelet count = 101000/mm^3^, and CRP at 496 mg/l. Aspartate aminotransferase, alanine aminotransferase, alkaline phosphatase, and bilirubin values were at the normal range.

An abdominal CT scan with contrast was obtained showing an acute pancreatitis grade E with two fluid collections in the pancreas. There was no evidence for gallstones. He was aggressively hydrated and received IV insulin.

A repeat abdomen CT scan, performed 7 days later, showed a necrotic fluid collection which was infected. An endoscopic drainage was performed. The patient's neurological status deteriorated with no sign of waking up and multiple episodes of seizures. A brain CT scan was performed, showing cortical and subcortical hypodense lesion in the right frontal area.

During his ICU stay, the patient developed septic shock with multiorgan failure. He died 45 days after ICU admission.

### 2.4. Case 4

A 42-year-old man with no significant medical history, presented after 10 days of polyuria and polydipsia, was admitted into the emergency department for vomiting and abdominal pain. No history of alcohol consumption was found.

The physical exam showed a diffuse abdominal tenderness. His blood pressure was at 100/70 mmHg, pulse at 130 beats/min, temperature at 37°C, and respiratory rate at 32 beats/min.

The laboratory tests showed serum glucose level at 23 mmol/l, hemoglobin A1c at 16.5%, pH = 7.26, bicarbonate = 7 mmol/l, anion gap = 36, serum sodium = 143 mmol/l, serum potassium = 2.9 mmol/l, lipase = 441 U/l, amylase = 361 U/l, cholesterol = 12.8 mmol/l, and triglyceridemia = 53 mmol/l with apparent lipema in the lab test tubes. WBC count was at 9200/mm^3^, platelet count at 153000/mm^3^, CRP at 330 mg/l, AST = 35 IU/l, ALT = 28 IU/l, bilirubin = 26 mmol/l, and the alkaline phosphatase=120 IU/l.

Computed tomography (CT) scan revealed an acute pancreatitis grade E with two fluid collections in the pancreas associated with peritoneal effusion. Fluid bolus and continuous insulin infusion were initiated. The patient's health state improved, and he was discharged after seven days.

All patient characteristics and laboratory results on admission are summarized in Tables 1 and 2, respectively. Figure 1 shows the evolution of triglyceride and blood glucose levels under treatment in all studied patients.

## 3. Discussion

Our cases confirm that AP can be induced by diabetic ketoacidosis (DKA) complicated by hypertriglyceridemia apart from any biliary cause or gallstone disease.

The literature analysis showed that the association between diabetic ketoacidosis, hypertriglyceridemia, and acute pancreatitis has rarely been reported. In fact, the triad of DKA with hypertriglyceridemia-induced acute pancreatitis occurs in 4% of cases [2]. It can affect both adults and children, as in our cases.

As far as the physiological side is concerned, there is a bidirectional relationship between dysglycemia and pancreatitis [4]. Acute pancreatitis could induce a beta cell dysfunction and transient insulin deficiency, and it could also increase insulin resistance due to systemic inflammation [5].

In this case, glycemic control can be achieved after remission of systemic inflammatory response and insulin infusion.

On the other hand, diabetes increases the risk of hypertriglyceridemia (HTG) which can induce acute pancreatitis. The mechanism by which HTG causes AP is still not completely understood. Many animal models have yielded some theories [6–8]. The first theory postulates that pancreatic lipase hydrolyses excess TG with the accumulation of free fatty acids in the pancreas. Free fatty acids then cause acinar cell and pancreatic capillary injury. The resultant ischemia creates an acidic environment, which further enhances free fatty acid toxicity [6].

The second hypothesis suggests that the hyperviscosity which is due to elevated levels of chylomicrons in these pancreatic capillaries leads to ischemia. HTG has also been shown to contribute to and accelerate the inflammatory cascade in animal models of AP [6].

The triad of acute pancreatitis (AP) coexisting with diabetes ketoacidosis (DKA) and hypertriglyceridemia (HTG) has a worse prognosis than a simple AP. In fact, this triad has the potential for devastating consequences with multiorgan failure. Many studies seek to evaluate AP outcomes in patients with and without DKA. According to Tsuang et al.'s analysis [6], the DKA group had significantly higher inpatient mortality compared to non-DKA-patients. DKA patients had almost 5 times higher odds of having AKI than those without DKA. Moreover, DKA subjects have a higher risk of ileus, shock, ARDS/mechanical ventilation, and/or SIRS [6]. They also need more parenteral nutrition, higher hospital charges, and longer in-hospital stay [2]. Other studies confirmed that the development of organ dysfunction and necrosis was present, more often observed in the hypertriglyceridemia-induced acute pancreatitis group than in the non-hypertriglyceridemia-induced acute pancreatitis group [2]. Making an early diagnosis is difficult due to overlapping signs, but it is important for a good recovery. In our study, all 4 patients were managed with fluid repletion and intravenous insulin infusion. Lipid-lowering treatments were started to decrease serum triglycerides. In our cases, 2 patients recovered relatively quickly, while 2 others died because they had severe pancreatitis and multiorgan failure, sepsis, and acute respiratory distress syndrome.

In our third case, the patient developed severe neurological signs with seizures. Pancreatic encephalopathy (PE) is very plausible. PE is a serious complication of severe acute pancreatitis, and it is considered a poor prognosis sign. The pathogenesis is not well elucidated. Many factors can be included: pancreatin activation, excessive release of cytokines and oxygen free radicals, microcirculation abnormalities of hemodynamic disturbance, ET-1/NO ratio, hypoxemia, bacterial infection, water and electrolyte imbalance, and vitamin B1 deficiency [7]. Understanding these factors is very important because it is the key to the prevention and best management of PE.

## 4. Conclusion

HTG-AP is associated with more complications and poor outcomes. The published studies concerning the mechanism of HTG-AP are in slow progress worldwide. Prospective ones enrolling a larger number of patients are still warranted to have more knowledge about HTG-AP and to promote the development of its therapeutic strategies.

## Figures and Tables

**Table 1:** Patients' characteristics.

	Case 1	Case 2	Case 3	Case 4
Age (years)	12	12	37	42
SAPSII in admission (points)	39	45	75	68
SOFA in admission (points)	16	20	18	16
ICU stay (days)	5	12	45	7
Mechanical ventilation (days)	0	12	45	0

**Table 2:** Laboratory results on ICU admission.

	Case 1	Case 2	Case 3	Case 4
Glucose serum (mmol/l)	24	21	32	42
Triglyceride (mmol/l)	133	53	75	53
Lipase (IU/l)	260	411	511	441
WBC/mm^3^	14650	21500	3900	9200
Platelet/mm^3^	88940	65000	101000	153000
pH	7.19	7.24	7.31	7.26
HCO_3_^−^ (mmol/l)	10	5	20.7	7
PaO_2_ (mmHg)	65	75	79	62
PaCO_2_ (mmHg)	12	14	45	20
PaO_2_/FiO_2_ (%)	309	150	131	88
Serum sodium (mmol/l)	135	142	132	143
Serum potassium (mmol/l)	2.5	2.7	3.9	2.9
Chloride (mmol/l)	98	88	99	87
CRP (mg/l)	350	214	496	330
